# Serum levels of ghrelin and LEAP2 in patients with type 2 diabetes mellitus: correlation with circulating glucose and lipids

**DOI:** 10.1530/EC-22-0012

**Published:** 2022-04-26

**Authors:** Jiaxi Li, Pu Huang, Jing Xiong, Xinyue Liang, Mei Li, Hao Ke, Chunli Chen, Yang Han, Yanhong Huang, Yan Zhou, Ziqiang Luo, Dandan Feng, Chen Chen

**Affiliations:** 1 Department of Physiology, Xiangya School of Medicine, Central South University, Changsha, Hunan, China; 2 Department of Health Management Center, Changsha Central Hospital, University of South China, Changsha, Hunan, China; 3 Department of Endocrinology, Xiangya Third Hospital, Central South University, Changsha, Hunan, China; 4 Department of Dermatology, Xiangya Third Hospital, Central South University, Changsha, Hunan, China; 5 Hunan Key Laboratory of Organ Fibrosis, Central South University, Changsha, Hunan, China; 6 School of Biomedical Sciences, University of Queensland, St Lucia, Brisbane, Queensland, Australia

**Keywords:** ghrelin, T2D, glucose, LEAP2, correlation

## Abstract

**Objective:**

Ghrelin regulates body weight, food intake, and blood glucose. It also regulates insulin secretion from pancreatic islet cells. LEAP2 is a newly discovered endogenous ligand of the growth hormone secretagogue’s receptor (GHSR). It not only antagonizes the stimulation of GHSR by ghrelin but also inhibits the constitutive activation of GHSR as an inverse agonist. Type 2 diabetes (T2D) patients have endocrine disorders with metabolic imbalance. Plasma levels of ghrelin and LEAP2 may be changed in obese and T2D patients. However, there is no report yet on circulating LEAP2 levels or ghrelin/LEAP2 ratio in T2D patients. In this study, fasting serum ghrelin and LEAP2 levels in healthy adults and T2D patients were assessed to clarify the association of two hormones with different clinical anthropometric and metabolic parameters.

**Design:**

A total of 16 females and 40 males, ages 23–68 years old normal (*n*  = 27), and T2D patients (*n*  = 29) were enrolled as a cross-sectional cohort.

**Results:**

Serum levels of ghrelin were lower but serum levels of LEAP2 were higher in T2D patients. Ghrelin levels were positively correlated with fasting serum insulin levels and HOMA-IR in healthy adults. LEAP2 levels were positively correlated with age and hemoglobin A1c (HbA1c) in all tested samples. Ghrelin/LEAP2 ratio was negatively correlated with age, fasting blood glucose, and HbA1c.

**Conclusions:**

This study demonstrated a decrease in serum ghrelin levels and an increase in serum LEAP2 levels in T2D patients. LEAP2 levels were positively correlated with HbA1c, suggesting that LEAP2 was associated with T2D development. The ghrelin/LEAP2 ratio was closely associated with glycemic control in T2D patients showing a negative correlation with glucose and HbA1c.

## Introduction

Ghrelin is a gastrointestinal tract-derived hormone containing 28 amino acids. It was first discovered in 1999 as an endogenous growth hormone secretagogue (GHS) to promote the secretion of GH (1). According to its acylation state by ghrelin O-acetyltransferase, ghrelin is divided into acyl ghrelin and des-acyl ghrelin. The ratio of two is 1:10 in plasma and 2:1 in the stomach (2). In adults, ghrelin is mainly produced by gastrointestinal endocrine cells (1). In addition, to stimulate GH release, ghrelin regulates food intake, body weight, and blood glucose by acting on CNS neurons and peripheral cells expressing GHSR1a to regulate the body’s response to changing metabolic states (3). Ghrelin also promotes gastrointestinal movement, induces gastric acid release, and produces antidepressants and other effects (4). In humans and rodents, plasma ghrelin increases during fasting and decreases during obesity. Plasma ghrelin levels also change dynamically under the influence of feeding state, with a rising before meal, and a rapid declining after meal (5). The increase in GH secretion by ghrelin may prevent hypoglycemia under hunger or calorie restriction (CR) (6, 7, 8). Early studies demonstrated that acyl ghrelin enhanced glucose-stimulated insulin secretion (GSIS) in human and rat islets *in vitro* (9, 10, 11). However, most recent studies in different systems and species strongly suggested an inhibitory role of ghrelin on GSIS (12, 13, 14, 15).

Recently (16, 17), liver expressed antimicrobial peptide 2 (LEAP2) was identified as an endogenous ligand of GHSR1a. LEAP2 was a secretory peptide first isolated from human blood in 2003 (18). The mature form of human LEAP2 contains 40 amino acids with two disulfide bonds and is primarily secreted from the liver and jejunum (16). As a key member of the ghrelin system, LEAP2 is the second endogenous ligand of GHSR. After binding with GHSR, LEAP2 not only prevents the activation of GHSR by ghrelin but also inhibits the constitutive activity of GHSR (19). Like ghrelin, plasma LEAP2 levels are highly regulated by body weight and feeding status. So far, most studies in humans and mice demonstrated opposite changes in LEAP2 and ghrelin in plasma. LEAP2 levels decreased in fasting and returned to baseline after refeeding (20). The roles of LEAP2 in glucose metabolism and insulin secretion are not clear yet. According to the existing results, LEAP2 was associated with glucose homeostasis. After oral administration of glucose in mice, the plasma LEAP2 levels were elevated. In addition, the levels of LEAP2 in plasma were higher in type 1 diabetic mice during CR (20); and the blood glucose levels were reduced by over-expression of LEAP2 in mice (16). In rat islet cells *in vitro*, the co-treatment of N-terminal LEAP2_21-12_ and ghrelin prevented the inhibitory effect of ghrelin on insulin secretion, suggesting that the N-terminal LEAP2 fragment blocked the action of ghrelin on GHSR (21). The latest research found that in cultured human islet cells, 1 μM LEAP2_38-47_ significantly enhanced GSIS (22).

Human plasma ghrelin levels were reduced in obese patients and negatively correlated with BMI in adults and children (23, 24, 25, 26, 27). It was reported that plasma ghrelin levels were decreased in T2D patients and closely associated with the decrease in the number of ghrelin cells in the pancreatic islets (12). In obese or overweight children, plasma LEAP2 levels were also reduced and negatively correlated with BMI and birth weight (28). In adults, obesity was associated with an increase in plasma LEAP2 levels and a decrease in plasma acyl ghrelin levels. Fasting plasma LEAP2 levels were positively correlated with obese-related metabolic parameters such as BMI, percentage of body fat, fasting blood glucose, and triglyceride (20). To our knowledge, this study is the first one that investigated the LEAP2 level and ghrelin/LEAP2 ratio in serum of T2D patients and normal controls. Because LEAP2 is an endogenous antagonist of GHSR, both ghrelin and LEAP2 are closely related to glucose metabolism and insulin secretion. It is clinically important to observe the dynamic changes of ghrelin and LEAP2 in T2D patients. The ratio of Ghrelin/LEAP2 may provide another useful index of glycemic control and diabetic progress. Therefore, the purpose of this study is to measure serum ghrelin and LEAP2 levels in T2D patients and to evaluate their correlations with age, gender, blood glucose, and lipids. It is hopeful to provide new targets to further investigation into T2D diagnosis, prognosis, and treatment.

## Methods

### Study design

The study was approved by the medical ethics committee of Changsha Central Hospital and carried out in accordance with the guidelines of the Helsinki Declaration and the legal provisions of China on human clinical research. Written informed consent and assent were obtained from all participants, as appropriate. Participants were recruited from the Department of Endocrinology, Changsha Central Hospital from October 2020 to June 2021.

### Study participants

The cross-sectional cohort consisted of 56 adults aged 23–68 years, including 27 healthy controls and 29 patients with T2D. The diagnosis of T2D is based on WHO's diagnostic criteria for diabetes in 2003. There was no history of any treatment for bile acids and lipids in the past 3 months, and no other types of diabetes, severe cardiovascular and cerebrovascular complications, liver and biliary obstructive diseases, chronic liver disease, chronic renal insufficiency, diarrhea, tumor, thyroid-related diseases, alcoholism, and not in pregnancy. Healthy adults were from the health management center of Changsha Central Hospital, Hunan Province, through routine health checks without any metabolic diseases in the last three consecutive years. There were no personal and family history of diabetes mellitus, hepatobiliary obstruction, hypertension, thyroid disease, autoimmune diseases, diarrhea, and tumor. They served as a healthy control group with normal fasting and postprandial blood glucose, hemoglobin A1c (HbA1c), liver, and kidney function. The weight and height of the subjects were measured and recorded. BMI was calculated by the formula weight divided by the square of height (kg/m^2^).

### Blood samples

The fasting blood samples were collected in the morning from 09:00 to 11:00 h with EDTA (1 mg/mL final) anticoagulation to separate plasma for the measurement of glucose, HbA1c, total cholesterol (TC), HDL-C, LDL-C, and triacylglycerols (TG). For insulin, ghrelin, and LEAP2 measurement, separate blood samples were collected without anticoagulation to obtain serum for ELISA assay. All blood samples were centrifuged at 3000 ***g*** for 10 min at 4°C to obtain plasma or serum. All samples were immediately stored at −80°C before the measurement in duplication in one run.

### Laboratory analyses

Blood glucose, HbA1c, TC, HDL-C, LDL-C, and TG levels were measured using standard enzymatic procedures in a Hitachi 7000 biochemical analyzer (Hitachi, Japan). Insulin, ghrelin, and LEAP2 were determined by specific ELISA (CSB-E05069h, CSB-E13398h, CSB-EL012853HU, respectively, Wuhan Huamei Biotech Co., Ltd, China). This ghrelin ELISA assay kit has been used in many human samples (29, 30, 31). In addition, this LEAP2 ELISA assay kit has been recently used in human hepatocyte samples (32). The assay kit has been validated by the manufacturer with specificity and sensitivity testing results in the information document. Assays were performed according to the manufacturer's instructions. Briefly, an antibody specific for LEAP2 was pre-coated onto a microplate. Standards and samples were pipetted into the wells and all LEAP2 in samples was bound by the immobilized antibody. After removing any unbound substances, a biotin-conjugated antibody specific for LEAP2 was added to the wells. After washing, avidin conjugated Horseradish Peroxidase (HRP) was added to the wells. Following a wash to remove any unbound avidin-enzyme reagent, a substrate solution was added to the wells and color develops in proportion to the amount of LEAP2 bound in the initial step. The color development was stopped, and the intensity of the color was measured at 450 nm using a microplate reader (Thermo Fisher Scientific). The fasting insulin levels and the homeostasis model assessment for insulin resistance (HOMA-IR = fasting glucose (mmol/L) × fasting insulin (µIU/mL) /22.5) were used as the index of insulin resistance (33). The concentrations of insulin, ghrelin, and LEAP2 were calculated by the logarithmic fitting of Microsoft Excel analysis software. Ghrelin/LEAP2 ratio was calculated by dividing ghrelin concentration (ng/mL) by LEAP2 concentration (ng/mL).

### Statistics and data analyses

The Shapiro–Wilk test was applied to determine whether variables had a normal distribution. The normally distributed variables were summarized as mean ± s.e.m., and nonparametric data were presented as median (interquartile range (IQR)). Since some variables were positively skewed, they were log-transformed (glucose, TG, ghrelin, and ghrelin/LEAP2) for all subsequent analyses and expressed as mean (95% CI). The difference between the two variables or nonparametric data was evaluated by the two-tailed Student’s t-testor Mann–Whitney test, respectively. Correlations were evaluated by the univariate and multivariate regression analysis. All statistical analyses were performed using Prism (version 7.0; GraphPad Software). A level of *P* < 0.05 was accepted as statistically significant.

## Results

### Characteristics of study subjects

Among all 56 subjects, 27 (48%) and 29 (52%) adults met the fasting blood glucose and HbA1c levels of normal or T2D, respectively. Among them, HbA1c levels of T2D patients were all above 6%. The subjects included 40 men and 16 women, accounting for 71% and 29% of the total respectively. Table 1 shows some basic characteristics of the participant subjects. The blood glucose and HbA1c levels of T2D patients were significantly higher than those of healthy individuals (*P* < 0.0001). HOMA-IR was also increased in the T2D group significantly (*P* < 0.0001), suggesting that T2D patients had an obvious insulin resistance. There was no significant difference in fasting serum insulin between the two groups (*P* = 0.6574). In addition, the T2D patients had higher average age, low HDL-C levels (*P* < 0.0001), and slightly higher LDL-C levels (*P* = 0.1107).

**Table 1:** Basic characteristics of healthy adults and T2D patients (*n*  = 27, 29). Data are presented as mean ± S.E.M. for age, weight, height, BMI, HbA1c, total cholesterol, HDL-C, and LDL-C. Glucose and TG are presented as mean (95% CI). Insulin and HOMA-IR are presented as median (IQR).

	Normal	T2D	*P* value
Age (years)	49.22 ± 1.775	54.28 ± 2.088	0.0727
Weight (kg)	66.37 ± 2.078	66.60 ± 1.703	0.9318
Height (cm)	166.4 ± 1.165	166.8 ± 1.292	0.8192
BMI	24.04 ± 0.660	23.92 ± 0.560	0.8866
Glucose (mmol/L)	** 5.0 (4.79; 5.20)**	** 9.3 (7.83; 10.77)**	<0.0001
Insulin (µIU/mL)	7.25 (6.79; 8.21)	7.47 (6.22; 8.52)	0.6574
HOMA-IR	**1.63 (1.41; 2.01)**	**3.02 (1.97; 4.30)**	<0.0001
HbA1c (%)	4.7 ± 0.077	7.2 ± 0.207	<0.0001
Total cholesterol (mg/dL)	4.9 ± 0.153	4.67 ± 0.215	0.3838
HDL-C (mg/dL)	1.27 ± 0.033	1.02 ± 0.044	<0.0001
LDL-C (mg/dL)	2.27 ± 0.261	2.76 ± 0.164	0.1107
TG (mg/dL)	1.92 (1.57; 2.27)	2.08 (1.27; 2.89)	0.3678

Bold values in the table are statistically significant (*P* < 0.05).

HbA1c, hemoglobin A1c; HOMA-IR, Homeostatic Model Assessment for Insulin Resistance; TG, triglycerides; T2D, type 2 diabetes.

### Serum levels of ghrelin and LEAP2

Table 2 shows serum levels of ghrelin and LEAP2, as well as the ghrelin/LEAP2 ratio of healthy adults and T2D patients in the male and female groups separately. Compared with healthy adults, serum ghrelin levels were decreased in T2D patients significantly, while LEAP2 levels were increased significantly. For gender differences, serum ghrelin levels were higher in females in both healthy and T2D groups. Compared with healthy adults, the ghrelin/LEAP2 ratio was significantly decreased in T2D patients in both female and male groups.

**Table 2:** Serum levels of LEAP2, ghrelin, ghrelin/LEAP2 ratio in healthy adults and T2D subjects, and in male and female groups (normal *n*  = 27: 19M+8F; T2DM *n*  = 29: 21M+8F). Data are presented as median (IQR) for LEAP2. Ghrelin and ghrelin/LEAP2 are presented as mean (95% CI).

	Normal			T2D			*P* value
	All	Male	Female	All	Male	Female	
LEAP2 (ng/mL)	1.61 (1.35; 2.23)	1.59 (1.32; 2.13)	1.83 (1.43; 2.58)	3.55 (1.64; 5.90)	3.37 (1.64; 5.90)	3.40 (1.28; 6.08)	**0.0080**
Ghrelin (ng/mL)	50.88 (41.57; 60.20)	47.54 (37.02; 58.05)	58.83 (36.17; 81.49)	37.46 (31.74; 43.17)	34.68 (29.57; 39.79)	44.75 (26.85; 62.65)	**0.0296**
Ghrelin/LEAP2	30.22 (23.14; 37.31)	29.96 (21.68; 38.24)	30.85 (13.47; 48.23)	14.60 (10.63; 18.57)	18.41 (12.54; 24.28)	17.05 (6.79; 27.30)	**0.0005**

Bold values are statistically significant (*P* < 0.05).

LEAP2, liver-expressed antimicrobial peptide 2; T2D, type 2 diabetes.

### Correlation analyses

Variables shown in Table 1 were subjected to correlation analyses with variables shown in Table 2. Results of correlation analyses were shown in Table 3. When all subjects were analyzed together, ghrelin levels did not correlate with any assessed variable. In contrast, LEAP2 levels and ghrelin/LEAP2 ratios were significantly correlated with different variables. In particular, LEAP2 levels were positively correlated with age and HbA1c levels. Ghrelin/LEAP2 ratio was negatively correlated with age, blood glucose, and HbA1c levels. For blood lipid parameters, no significant correlation was found except for the ghrelin/LEAP2 ratio, which was negatively correlated with LDL-C levels. No significant correlation was found between ghrelin, LEAP2, or ghrelin/LEAP2 ratio and BMI, insulin, HOMA-IR, total cholesterol, HDL-C, or TG.

**Table 3:** Correlations between LEAP2, ghrelin, ghrelin/LEAP2 with age, BMI, glucose, insulin, HOMA-IR, HbA1c, total cholesterol, HDL-C, LDL-C, and TG in all the subjects, in healthy adults, and in diabetics (*n*  = 27, 29).

	Group	Ghrelin	LEAP2	Ghrelin/LEAP2
Age (years)	All	−0.106	**0.398**	−0.385
	Normal	−0.093	0.325	−0.362
	T2D	0.044	**0.371**	−0.310
BMI (kg/m^2^)	All	0.082	0.051	−0.075
	Normal	0.228	0.181	0.008
	T2D	−0.163	0.021	−0.290
Glucose (mmol/L)	All	−0.145	0.230	−0.318
	Normal	0.293	−0.144	0.210
	T2D	0.082	0.028	−0.110
Insulin (µIU/mL)	All	0.061	−0.058	−0.043
	Normal	**0.4791**	0.1151	0.1852
	T2D	−0.075	−0.141	−0.089
HOMA-IR	All	−0.082	0.085	−0.236
	Normal	**0.489**	0.063	0.203
	T2D	−0.037	−0.107	−0.129
HbA1c (%)	All	−0.218	**0.376**	−0.459
	Normal	0.017	0.224	−0.148
	T2D	0.281	0.141	−0.105
Total cholesterol (mg/dL)	All	−0.008	0.198	−0.162
	Normal	−0.056	**0.585**	−0.477
	T2D	−0.052	0.180	−0.059
HDL-C (mg/dL)	All	0.226	−0.044	0.214
	Normal	0.214	0.131	0.157
	T2D	−0.079	0.195	−0.300
LDL-C (mg/dL)	All	−0.151	0.171	−0.377
	Normal	0.014	**0.537**	−0.451
	T2D	−0.313	−0.116	0.003
TG (mg/dL)	All	0.087	−0.032	0.016
	Normal	0.067	0.158	−0.128
	T2D	0.174	−0.087	0.179

Bold values are statistically significant (*P* < 0.05).

HOMA-IR, Homeostatic Model Assessment for Insulin Resistance; HbA1c, hemoglobin A1c; TG, triglycerides; T2D, type 2 diabetes.

In the correlation analysis described above, the subjects were divided into healthy adults and T2D groups (Table 3). Ghrelin levels were positively correlated with fasting serum insulin levels (*r* = 0.4791, *P* = 0.0114) and HOMA-IR (*r* = 0.4886, *P* = 0.0097) in healthy adults only. LEAP2 levels were positively correlated with age and HbA1c in all subjects and T2D patients, respectively. Ghrelin/LEAP2 ratio was negatively correlated with age in healthy adults. For blood lipid parameters, LEAP2 levels were positively correlated with plasma total cholesterol levels, and the correlation was stronger in healthy adults than that in T2D patients (*r* = 0.5847, *P* = 0.0014). LEAP2 levels were positively correlated with LDL-C in the healthy adult group (*r* = 0.5368, *P* = 0.0039). Ghrelin/LEAP2 ratio was negatively correlated with plasma total cholesterol and LDL-C in healthy adults. There was no significant correlation between ghrelin, LEAP2, or Ghrelin/LEAP2 ratio and BMI, HDL-C, or TG.

Correlation analyses of the variables were separately conducted on male and female adults (Table 4). Surprisingly, ghrelin levels were negatively correlated with HbA1c levels only in male individuals. Ghrelin levels did not correlate with any other variables in male or female adults. LEAP2 levels were positively correlated with age and HbA1c levels in both male and female adults, but the correlation was stronger in females than that in male adults. LEAP2 levels were positively correlated with blood glucose levels only in the female group. Ghrelin/LEAP2 ratio was negatively correlated with age, and the correlation was stronger in females than that in male individuals. Ghrelin/LEAP2 ratio was negatively correlated with blood glucose and HbA1c levels only in male individuals. For blood lipid parameters, no significant correlations were found between ghrelin levels and any blood lipid parameter. LEAP2 levels were positively correlated with TG levels only in female individuals. Ghrelin/LEAP2 ratio was negatively correlated with LDL-C levels only in male individuals (*r* = −0.4705, *P* = 0.0022). Similarly, BMI and LDL-C were not significantly correlated with all assessed variables between male and female groups.

**Table 4:** Correlations between LEAP2, ghrelin, ghrelin/LEAP2 with age, BMI, glucose, insulin, HOMA-IR, HbA1c, total cholesterol, HDL-C, LDL-C, and TG in all the subjects, in male adults, and in female adults (*n*  = 40, 16).

	Group	Ghrelin	LEAP2	Ghrelin/LEAP2
Age (years)	All	−0.106	**0.398**	−0.385
	Male	−0.060	**0.405**	−0.285
	Female	−0.210	0.406	−0.617
BMI (kg/m^2^)	All	0.082	0.051	−0.075
	Male	0.183	−0.014	0.001
	Female	0.054	0.267	−0.193
Glucose (mmol/L)	All	−0.145	0.230	−0.318
	Male	−0.303	0.141	−0.331
	Female	0.138	**0.549**	−0.283
Insulin (µIU/mL)	All	0.061	−0.058	−0.043
	Male	0.128	−0.060	−0.051
	Female	−0.058	−0.060	0.006
HOMA-IR	All	−0.082	0.085	−0.236
	Male	−0.088	0.028	−0.227
	Female	0.034	0.472	−0.287
HbA1c (%)	All	−0.218	**0.376**	−0.459
	Male	−0.340	**0.343**	−0.473
	Female	−0.061	**0.507**	−0.426
Total cholesterol (mg/dL)	All	−0.008	0.198	−0.162
	Male	−0.183	0.166	−0.109
	Female	0.124	0.371	0.314
HDL-C (mg/dL)	All	0.226	−0.044	0.214
	Male	0.181	−0.026	0.206
	Female	0.208	−0.097	0.204
LDL-C (mg/dL)	All	−0.151	0.171	−0.377
	Male	−0.294	0.135	−0.471
	Female	0.046	0.310	−0.213
TG (mg/dL)	All	0.087	−0.032	0.016
	Male	0.005	−0.154	0.128
	Female	0.418	**0.702**	−0.370

Bold values are statistically significant (*P* < 0.05).

HOMA-IR, Homeostatic Model Assessment for Insulin Resistance; HbA1c, hemoglobin A1c TG, triglycerides.

## Discussion

Here, we assessed serum levels of ghrelin and LEAP2 in healthy adults and T2D patients. The correlation between each hormone and anthropometric and metabolic-related parameters was investigated. To our knowledge, this is the first clinical study of circulating levels of LEAP2, an endogenous antagonist and inverse agonist of GHSR, in T2D patients. In addition, the change of ghrelin/LEAP2 ratio in T2D patients was revealed to reflect diabetic and metabolic parameters.

As expected, T2D patients had higher fasting blood glucose and HbA1c levels, and a higher HOMA-IR index in this study. HbA1c levels reflect well the long-term glycemic control. In addition, T2D patients had lower HDL-C levels and higher LDL-C levels, although no significant difference was reached in LDL-C levels (*P* = 0.1107). These indicators reflected the basic characteristics of T2D: a continuous elevation of blood glucose led to the disturbance of lipid metabolism and hyperlipidemia. These results were consistent with the occurrence of complications in T2D patients, whose blood glucose levels were not well controlled with lipid metabolic disorders (34).

Through data analysis, T2D patients had lower circulating ghrelin levels than that in healthy individuals. The serum ghrelin levels in males were lower than that in females, although this difference did not reach statistical significance. The ghrelin levels were positively correlated with fasting serum insulin and HOMA-IR in healthy adults, and negatively correlated with HbA1c levels in male individuals. No correlations were found between the ghrelin levels and other metabolic parameters measured in this study. Previous studies reported that ghrelin levels in obese, insulin-resistant, or metabolic syndrome patients were reduced (35, 36, 37), and such reduction of ghrelin contributed to the development of T2D (38, 39). In T2D patients, plasma ghrelin levels were reduced and associated with a decrease in the number of ghrelin cells in the pancreatic islets, leading to a possible decrease in local ghrelin levels in the islets (12). The plasma ghrelin levels were decreased in BMI >25 kg/m^2^ in overweight or BMI >30 kg/m^2^ in obese patients with T2D, suggesting that this decrease in ghrelin might be an adaptative response to positive energy balance in overweight and obese patients (23, 24, 40). However, ghrelin levels in the underweight individuals were reported to be very low, even lower than the already decreased ghrelin levels in obese individuals (41). The reason for such a depressed level of ghrelin in underweight individuals was not clear yet. Ghrelin levels were reported to be associated with glycemic controls. The T2D patients with poor glycemic control had lower ghrelin levels than that in patients with relatively good glycemic control (42). The HbA1c levels reflect well the glycemic control of patients in recent 3 months. Patients with abnormally elevated HbA1c values showed a lower ghrelin level in this study. The correlation between HbA1c and ghrelin levels was evident in male individuals only. In regard to gender difference reported here, it might be caused by the small number of female subjects in this study (16 cases) or the effects of female-specific endocrine profiles.

Many studies reported that plasma ghrelin levels were negatively correlated with BMI, insulin levels, and body fat in diabetic and non-diabetic patients (3, 4, 5). In the current study and other studies, there was no significant correlation between ghrelin levels and anthropometric or metabolic variables. These studies included non-diabetic adolescents and healthy adults (28, 43). In addition, earlier studies reported that exogenous ghrelin administration induced insulin resistance in rats and humans (44, 45, 46). Results in this study supported a correlation between ghrelin levels and insulin resistance. Interestingly, this positive correlation appeared only in healthy adults but not in T2D patients. In this study, ghrelin levels were negatively correlated with HbA1c levels in males and positively correlated with fasting insulin levels and HOMA-IR in healthy adults. Such reduction in ghrelin under diabetic conditions may be a physiologically adaptative response to reduce appetite and weight gain during insulin resistance and obesity.

In this study, LEAP2 levels in the circulation of T2D patients were measured. LEAP2 levels were increased significantly in T2D patients in this study, which was the opposite to the change in ghrelin levels. There are very few reports on LEAP2 levels in circulation to date, which may be related to its unclear physiological function yet *in vivo*. Among the available data, Fittipaldi reported that LEAP2 levels were decreased in overweight or obese adolescents (28). In contrast, Mani studied plasma LEAP2 levels in type 1 diabetic mice and patients and concluded that plasma LEAP2 levels were increased with increased body weight and blood glucose levels (20). Both oral glucose and type 1 diabetic hyperglycemia significantly increased plasma LEAP2 levels (20). There was a significant positive correlation between LEAP2 levels and blood glucose levels in mice (47). Mean plasma LEAP2 levels were significantly increased in adults with BMI between 25-40 kg/m^2^ and further increased in BMI greater than 40 kg/m^2^ (20). In addition, fasting plasma LEAP2 levels were positively correlated with obesity-associated metabolic indexes such as BMI, percentage of body fat, fasting blood glucose, and triglyceride levels (20). The plasma LEAP2 levels were reported to be positively correlated with age, BMI, and fasting insulin in patients with hepatic steatosis, but negatively correlated with acyl ghrelin (48). In this study, LEAP2 levels were positively correlated with age, blood glucose, and HbA1c levels, and the correlations were stronger in female than male individuals. Similarly, with a decrease in ghrelin, an increase in LEAP2 secretion may inhibit the stimulation of ghrelin on appetite and weight gain to maintain ‘normal’ energy homeostasis. For blood lipid indexes, LEAP2 levels were positively correlated with plasma total cholesterol and triglyceride levels of female individuals in this study, consistent with earlier reports. Gender differences in LEAP2 levels were reported in adolescent boys and girls showing higher levels in girls after puberty than that in boys or in girls before puberty. In addition, LEAP2 levels were positively correlated with insulin, IGF-1, and triglyceride levels in girls, but negatively correlated with ghrelin levels (49). The reason for such gender difference in LEAP2 is still unknown but such difference may suggest different roles in males and females.

The changes in serum ghrelin/LEAP2 ratio in healthy adults and T2D patients were evaluated in this study. We found ghrelin/LEAP2 ratio was significantly decreased in T2D patients. This is a consistent and amplified observation of the decrease in ghrelin levels and increase in LEAP2 levels in diabetic patients in this study. To our knowledge, this is the first report of ghrelin/LEAP2 ratio in relation to diabetes. It was reported that there was no significant difference in the ratio among normal weight, overweight, or obese adolescents (24). In this study, the ghrelin/LEAP2 ratio in T2D patients was reduced, and negatively correlated with age, blood glucose, and HbA1c levels. Gender differences existed too. For example, correlations between ghrelin/LEAP2 ratio and age were stronger in female individuals than that in male individuals. The negative correlation between ghrelin/LEAP2 ratio and blood glucose or HbA1c levels only appeared in male individuals. Functionally, the decrease in ghrelin/LEAP2 ratio in T2D patients may reduce the over-activation of GHSR in obesity to return to normal energy homeostasis. This view is supported by a report that obesity and diabetes were improved by reducing acyl ghrelin levels, increasing LEAP2 levels, or blocking GHSR activity (50). Therefore, we believe that the reduced serum ghrelin levels and increased LEAP2 levels in T2D patients may be a physiologically compensatory response to positive energy balance in order to maintain normal energy homeostasis. A recent report suggested that proteins regulating GHSR1a function represented a potentially exciting new therapeutic target to treat appetite dysfunction, Alzheimer’s disease, insulin deficiency, and chronic inflammation (51). GHSR is highly expressed in polypeptide cells in mouse and human pancreatic islets (52). Circulation ghrelin and LEAP2 levels may therefore play an important role in regulating pancreatic islet function, which may influence glycemic control through regulating insulin secretion.

In conclusion, ghrelin and LEAP2 levels in healthy adults and T2D patients were assessed. Serum ghrelin levels in T2D patients were reduced, while LEAP2 levels were elevated in this study, resulting in a significant decrease in the ghrelin/LEAP2 ratio. Combined action of ghrelin and LEAP2 may play an important role in the development of T2D. The ghrelin-LEAP2 axis may be a potential target for the treatment of T2D. In addition, ghrelin and LEAP2 levels are closely correlated with lipid metabolism, warranting future investigations into the mechanism of LEAP2 action.

## Declaration of interest

The authors declare that there is no conflict of interest that could be perceived as prejudicing the impartiality of the research reported.

## Funding

This work was funded by National Natural Science Foundation of China (81870059, 82070068 for Ziqiang Luo).

## Author contribution statement

D D F and J X L designed the study. J X L, P H, J X, X Y L, M L, H K, C L C, Y H, Y H H, Y Z collected, analysed and/or interpreted the data. J X L, D D F, Z Q L, C C wrote and edited the manuscript. All authors approved the submitted and published versions.
